# Life-threatening pulmonary artery rupture during transcatheter tricuspid valve-in-ring implantation: case report and management strategies

**DOI:** 10.1093/ehjcr/ytaf351

**Published:** 2025-08-16

**Authors:** Kadir Sadikoglu, Dilara Pay, Serkan Aslan, Gamze Babur Guler, Mehmet Erturk

**Affiliations:** Surgery Training and Research Hospital, Department of Cardiology, University of Health Sciences Istanbul Mehmet Akif Ersoy Thoracic and Cardiovascular, Turgut Ozal Bulvarı No:11, Istanbul, Kucukcekmece 34303, Turkey; Surgery Training and Research Hospital, Department of Cardiology, University of Health Sciences Istanbul Mehmet Akif Ersoy Thoracic and Cardiovascular, Turgut Ozal Bulvarı No:11, Istanbul, Kucukcekmece 34303, Turkey; Surgery Training and Research Hospital, Department of Cardiology, University of Health Sciences Istanbul Mehmet Akif Ersoy Thoracic and Cardiovascular, Turgut Ozal Bulvarı No:11, Istanbul, Kucukcekmece 34303, Turkey; Surgery Training and Research Hospital, Department of Cardiology, University of Health Sciences Istanbul Mehmet Akif Ersoy Thoracic and Cardiovascular, Turgut Ozal Bulvarı No:11, Istanbul, Kucukcekmece 34303, Turkey; Surgery Training and Research Hospital, Department of Cardiology, University of Health Sciences Istanbul Mehmet Akif Ersoy Thoracic and Cardiovascular, Turgut Ozal Bulvarı No:11, Istanbul, Kucukcekmece 34303, Turkey

**Keywords:** Pulmonary artery rupture, Transcatheter tricuspid valve-in-ring implantation, Tricuspid regurgitation, Vascular plug embolization, Case report

## Abstract

**Background:**

This case report highlights pulmonary artery rupture as a rare but severe complication during transcatheter valve-in-ring implantation (TVIRI). The case offers practical preventive strategies to enhance procedural safety in high-risk patients undergoing TVIRI.

**Case summary:**

A 54-year-old female patient presented with severe tricuspid regurgitation due to annuloplasty ring failure. The TVIRI was complicated by pulmonary artery rupture, resulting in massive haemoptysis and haemodynamic collapse. The bleeding was successfully controlled by prompt management with balloon tamponade and selective embolization using an Amplatzer Vascular Plug 4.

**Discussion:**

This case demonstrates the potential for life-threatening complications during TVIRI and highlights the importance of careful procedural planning, multidisciplinary collaboration, and prompt intervention to ensure patient safety.

Learning points

- Pulmonary artery rupture risk: transcatheter tricuspid valve-in-ring implantation procedures carry a rare but life-threatening risk of pulmonary artery rupture, requiring careful device handling and imaging.
- Prompt injury management: rapid identification of vascular injury and timely interventions, such as embolization or stent placement, are critical to prevent fatal outcomes.

## Introduction

Tricuspid regurgitation (TR) is a prevalent valvular heart disease that is associated with considerable morbidity and mortality. A surgical annuloplasty ring has been recognized as a conventional approach for managing severe TR.^1,2^ Nevertheless, there is an increasing recognition of ring failure and recurrent TR, particularly in the context of advanced heart failure and structural deterioration.^3^ In light of these observations, transcatheter valve-in-ring implantation (TVIRI) has emerged as a less invasive treatment option for these high-risk patients.^4^ Despite its growing adoption, this procedure is not without risks. Here, we present a case of pulmonary artery rupture, a rare but life-threatening complication during TVIRI implantation, and discuss its management and implications for clinical practice.

## Summary figure

**Figure:**
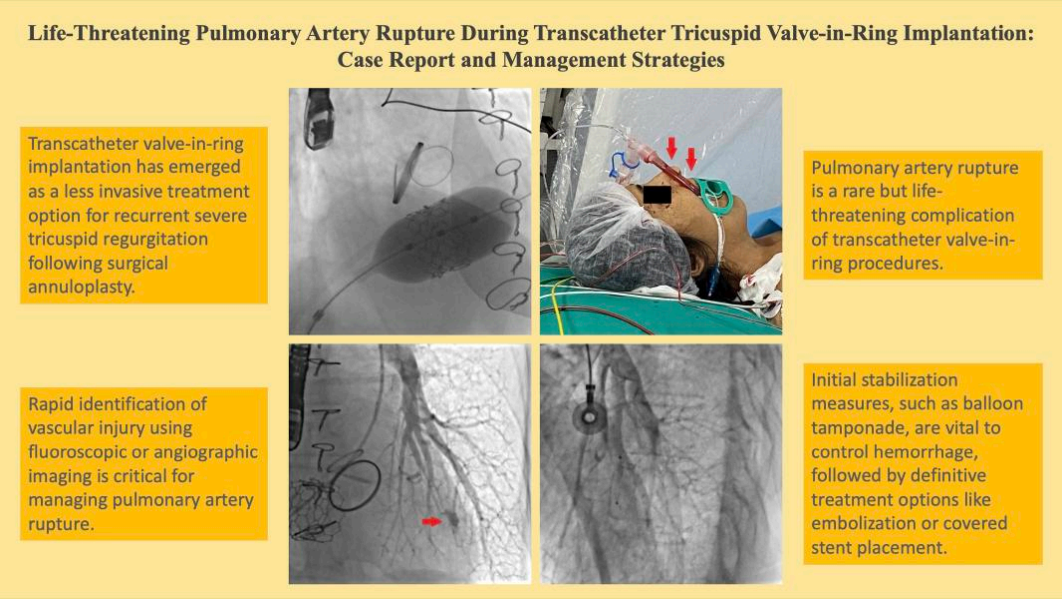


## Case report

A 54-year-old woman had history of end-stage renal disease on haemodialysis for the last two years, multiple myeloma currently in remission, and tricuspid annuloplasty with 29 mm Duran AnCore annuloplasty system (Medtronic, Inc., Minneapolis, MN, USA) concomitant with 29 mm mitral and 19 mm aortic St Jude Medical mechanical heart valves (St Jude Medical, Inc, St Paul, MN) for rheumatic in aetiology in 2019. A chronological overview of the patient’s presentation, intervention, and outcome is summarized in *Table 1*. She presented with progressive fatigue, dyspnoea on exertion, and signs of right-sided heart failure. On physical examination, her blood pressure was 102/68 mmHg, heart rate 92 b.p.m., and oxygen saturation 94% on room air. Cardiovascular exam revealed elevated jugular venous pressure, hepatomegaly, and peripheral oedema. Laboratory findings revealed haemoglobin 10.8 g/dL (normal: 11.7–15.5 g/dL), creatinine 4.6 mg/dL (normal: 0.6–1.1 mg/dL), and NT-proBNP 18 800 pg/mL (normal: 0–125 pg/mL). Transoesophageal echocardiography (TEE) revealed a dilated right ventricle, severely impaired systolic function, and torrential TR due to persistent leaflets malcoaptation with structural failure of the annuloplasty ring. Given her high surgical risk profile, a multidisciplinary heart team adopted for TVIRI. The size of the transcatheter heart valve (THV) was measured using the reference ring size and computed tomography measurements (diameters: 25.5 × 26.5 mm, area: 529 mm^2^, perimeter: 8 186 mm, area-derived diameter: 26 mm) and confirmed using the Valve-in-Valve Mitral app.^5^ Although a 29-mm SAPIEN 3 valve is commercially available, annular sizing (area 529 mm², perimeter 81.86 mm) suggested suboptimal fitting based on the Valve-in-Valve Mitral app recommendations. Consequently, a 30.5-mm Myval THV (Meril Life Sciences Pvt. Ltd., Gujarat, India) was selected due to its broader sizing options and expansion characteristics, which were considered favourable for sealing within the existing annuloplasty ring.

**Table 1:** Timeline of clinical presentation, management, and follow-up

Time point	Event
2019	Surgical implantation of tricuspid annuloplasty ring and mechanical valves
2024	Presentation with progressive right-sided heart failure symptoms
Day 0	Transcatheter tricuspid valve-in-ring implantation
Day 0 (intra-procedure)	Pulmonary artery rupture occurred
Day 0 (intra-procedure)	Balloon tamponade and AVP 4 deployment
Day 10	Discharged with stable haemodynamics
1-Month follow-up visit	NYHA Class II, no recurrence of haemoptysis
6-Month follow-up visit	Clinically stable, NYHA Class II, no recurrence of haemoptysis or valve dysfunction

AVP, Amplatzer Vascular Plug; NYHA, New York Heart Association.

**Figure 1:**
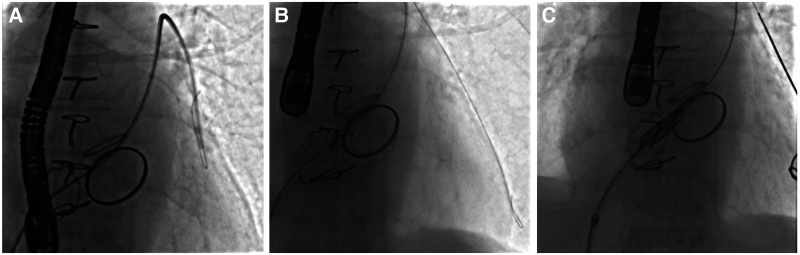
(*A*) The 6 F Judkins right (JR) 4 diagnostic catheter is passed through the tricuspid valve. (*B*) The hydrophilic guidewire is advanced 0.035 inches into the left pulmonary artery. (*C*) The 0.035-inch super-stiff guidewire is advanced through the JR 4 into the left pulmonary artery, and a 29-mm Myval transcatheter heart valve is placed over this wire to the level of the tricuspid valve.

**Figure 2:**
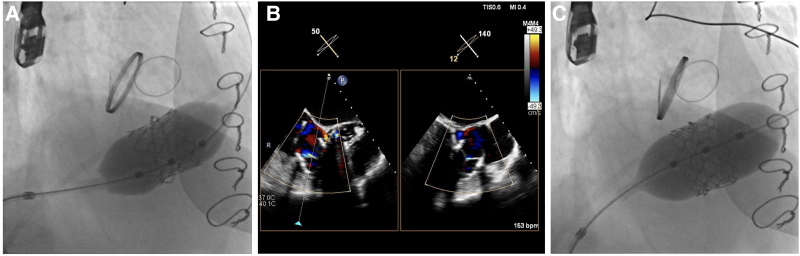
(*A*) Once the valve was correctly positioned, it was expanded to fit inside the tricuspid annuloplasty ring. (*B*) Control transoesophageal echocardiography showing a moderate paravalvular leak. (*C*) Post-dilation of the valve to reduce paravalvular leakage.

**Figure 3:**
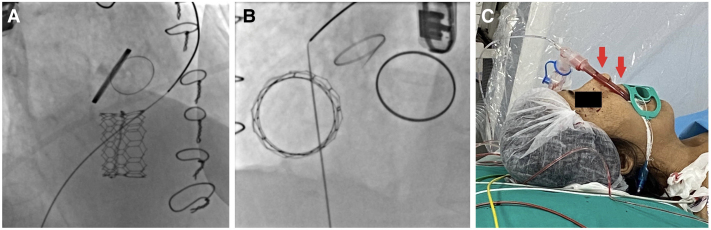
(*A*, *B*) Fluoroscopy confirmed that adequate annular expansion was achieved. (*C*) There was blood in the endotracheal tube at the end of the procedure (marked with arrows).

The procedure was performed under general anaesthesia with fluoroscopic and TEE guidance. A 6 F Judkins right (JR) 4 diagnostic catheter was used to cross the tricuspid valve and advanced through the right heart ventricle into the left pulmonary artery, using a 0.035-inch hydrophilic guidewire. A super-stiff guidewire (0.035-inch Back-up Meier 300-cm J-tip, Boston, USA) was then placed into the left pulmonary artery via the JR 4 in the correct position (*Figure 1A–C*, Video 1). The 30.5-mm Myval THV was put in position across the tricuspid valve over a super-stiff wire and was optimized aiming at a 20/80 right atrium to the right ventricle position. The valve was then slowly expanded with 2 mL overfilling, without rapid pacing (*Figure 2A*, Video 2). A control transoesophageal echocardiogram showed a paravalvular leak (*Figure 2B*). The valve was then post-dilated with the same balloon to 35 cc to reduce paravalvular leakage (*Figure 2C*). Fluoroscopy confirmed good circumferential THV apposition within the ring (*Figure 3A* and *B*). No paravalvular leak was observed.

**Figure 4:**
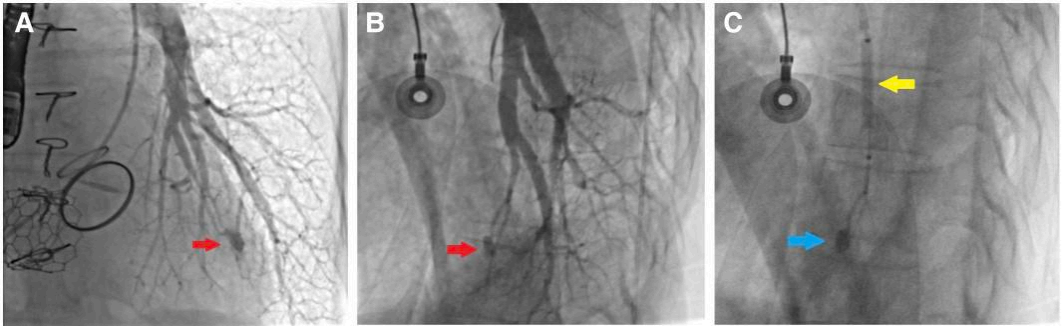
(*A*, *B*) There was a perforation of the main branch of the right pulmonary artery (red arrows). (*C*) The 4.0 × 40 mm peripheral balloon (yellow arrow) was inflated for 20 min. Nevertheless, this intervention did not lead to the cessation of bleeding (blue arrow).

**Figure 5:**
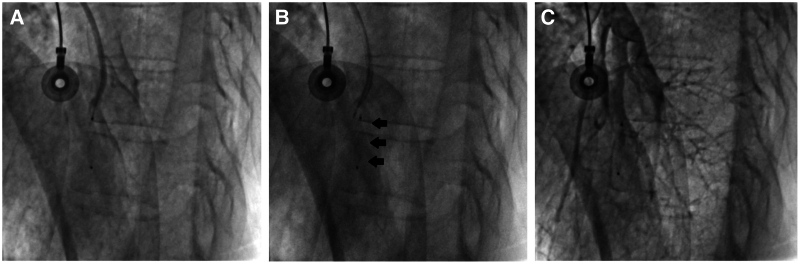
(*A–C*) Positioning of Amplatzer Vascular Plug 4 in a perforated pulmonary artery under fluoroscopic guidance.

Everything seemed perfect until the endotracheal tube was noticed. At the end of the procedure, blood was seen in the endotracheal tube (*Figure 3C*). Rapidly, haemoptysis became massive, leading to haemodynamic collapse. We hypothesized that the super-stiff wire caused localized trauma to the distal pulmonary artery. JR 4 diagnostic catheter was rapidly advanced until it reached the left pulmonary artery. Selective injection confirmed the perforation of a major branch of the left pulmonary artery (*Figure 4A* and *B*). A 0.035-inch hydrophilic wire was introduced into the left distal pulmonary artery and the 7 F JR guiding catheter was advanced into the suspected pulmonary branch over the wire. Initially, a 4.0 × 40 mm peripheral balloon was maintained in a state of inflation for a duration of 20 min, with the objective of interrupting the supply of blood to the bleeding artery and preventing the recurrence of haemoptysis (*Figure 4C*). However, this intervention did not result in the cessation of bleeding. Given the patient’s fragile condition, a decision was made to proceed with selective embolization of the ruptured artery rather than transferring the patient to the surgical suite, thereby conserving time and resources. For diameter measurement, visualization of images was conducted on quantitative angiography in different views (anterior–posterior and lateral views). The diameter of the injured pulmonary artery branch was measured using automated coronary angiography software. The calibration process was performed with reference to the catheter diameter. The diameter of the injured pulmonary artery branch was measured at 5 mm. The wire was then extracted, and an Amplatzer Vascular Plug 4 (6 × 11 mm) (Abbot, IL, USA) was strategically positioned and deployed under the guidance of fluoroscopic imaging (*Figure 5A–C*, Video 3). Control angiography confirmed the cessation of the bleeding. The patient was transferred to the intensive care unit with stable haemodynamic and respiratory conditions. The patient’s haemodynamics improved, and she was discharged on post-procedure Day 10 with optimized medical therapy. She was discharged on oral carvedilol 6.25 mg twice daily and warfarin 5 mg once daily. As the patient was anuric and receiving haemodialysis, diuretics such as furosemide and spironolactone were not prescribed. At the first and sixth month follow-up visits, she remained clinically stable with New York Heart Association (NYHA) Class II symptoms and no recurrence of haemoptysis or valve dysfunction.

## Discussion

Recent reports have emphasized the complexity of transcatheter interventions in tricuspid positions, highlighting the importance of multimodality imaging and preprocedural planning.^6–8^ Pulmonary artery rupture, though rare, has been described in right heart catheterization and structural interventions as a potentially fatal complication requiring immediate action.^9,10^

Pulmonary artery rupture during TVIRI, although rare, represents a catastrophic complication that demands prompt recognition and management. Vessel injury is associated with trauma to the pulmonary artery or its branches caused by the catheter itself, the wire, or the balloon.^11^

Several mechanisms may contribute to vascular injury in this setting. Excessive advancement of the super-stiff guidewire into small, distal branches of the pulmonary artery can result in perforation due to the fragile nature of these vessels. Unintentional forward migration of the guidewire during manipulation of the delivery system or valve positioning may exacerbate this risk, particularly if continuous fluoroscopic monitoring is not maintained. Furthermore, tension or straightening of the guidewire may generate shearing forces that can traumatize the vessel wall. In our case, a diagnostic JR catheter was utilized for valve crossing and wire exchange rather than a balloon-tipped catheter, which could have offered additional protection against distal vessel injury. The super-stiff Meier wire was advanced into a relatively distal branch without deliberately forming a J curve, which may have increased the risk of perforation. We have now recognized that adding a J curve to stiff wires and preferentially using a balloon-tipped catheter for initial crossing are important preventive steps to minimize deep distal engagement and vessel trauma.

To minimize the risk of pulmonary artery perforation during TVIRI, several preventive strategies should be considered. Operators should avoid positioning the guidewire into small, peripheral branches whenever possible, favouring more proximal and larger-calibre vessels.

Maintaining a gentle wire curvature and avoiding excessive tension are key principles. Continuous fluoroscopic monitoring is essential throughout critical procedural steps, including wire placement, device advancement, and valve deployment. Using a softer or less stiff guidewire during initial valve crossing may be considered in high-risk patients. Reassessing the wire position after each catheter manipulation is crucial to avoid inadvertent injury. Adherence to these preventive measures may substantially reduce the likelihood of guidewire-related vascular injury and improve procedural safety. However, in anatomically challenging cases, advancing the guidewire into more distal branches may offer improved support and stability for transcatheter valve delivery. Thus, the optimal wire position must balance procedural safety with the need for sufficient device support. In such situations, operators should carefully weigh the risks and benefits of distal wire placement, maintaining meticulous control under continuous fluoroscopic guidance.

In our case, a careful review of procedural recordings revealed that the guidewire tip was withdrawn during balloon expansion, accompanied by minor back-and-forth movements before and after valve deployment. It is plausible that these dynamic movements, particularly repositioning attempts to optimize valve alignment, may have traumatized the distal pulmonary artery branch. Such manipulations could have weakened the vessel wall, rendering it more susceptible to perforation upon additional mechanical stress. This observation underscores the critical importance of minimizing guidewire manipulation during valve deployment. Maintaining a stable wire position, ensuring continuous fluoroscopic monitoring, and avoiding excessive advancement or withdrawal of the wire during critical procedural steps are essential to prevent vessel injury. Regarding the delivery system, the Myval nose cone is designed to be relatively soft and atraumatic. Based on procedural review, no direct trauma from the delivery system itself was identified; rather, the vessel injury was attributed to wire-related trauma exacerbated by manipulation during valve deployment.

In cases of vascular injury, prompt recognition and management are vital. Collaboration with a multidisciplinary team, including interventional cardiologists, cardiothoracic surgeons, and anaesthesiologists, is essential for optimal outcomes. Initial steps include rapid identification of the bleeding source using fluoroscopic or angiographic imaging. In parallel with interventional treatments, adjunctive measures should be taken, including calling the anaesthesiologist and cardiac surgeon, resuscitation if needed, administration of intravenous fluids if hypotension ensues, blood transfusion, securing the airways (selective lung intubation in cases with suffocation), and reversing anticoagulation (protamine injection if heparin was given).

Management strategies for pulmonary artery or its branches rupture include immediate balloon tamponade to control bleeding, followed by embolization techniques such as the deployment of vascular plugs or coil embolization.^12–14^ Pulmonary lobectomy can be performed as an emergency life-saving solution when other techniques have failed.^14^ In this case, balloon tamponade was attempted initially, but persistent bleeding necessitated definitive embolization. The Amplatzer Vascular Plug 4 (AVP 4) was chosen over AVP 2 or 3 because of the small diameter (5 mm) and distal location of the injured vessel. The flexible, low-profile design of the AVP 4 allowed for deployment through a 7 F JR guiding catheter without requiring large sheath sizes, minimizing the risk of further trauma.^15^ Although coil embolization could have been considered, concerns about the need for multiple coils, prolonged manipulation, and incomplete haemostasis led us to favour the plug approach in a haemodynamically unstable patient. A single AVP 4 device achieved immediate occlusion and control of bleeding. One of the most widely accepted uses of the AVP is the embolization of pulmonary arteriovenous malformations.^16,17^ However, to the best of our knowledge, this case represents the first reported use of an AVP for the successful treatment of pulmonary artery perforation during TVIRI. This proactive approach enhances preparedness and ensures that life-threatening complications can be managed effectively in real time.

## Conclusion

The emergence of TVIRI represents a significant advancement in the management of recurrent TR following annuloplasty. However, awareness and preparedness for complications such as pulmonary artery rupture are crucial. Future developments in device design and procedural protocols may further enhance the safety and efficacy of these interventions.

## Lead author biography


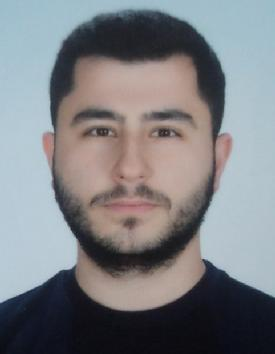


Kadir Sadikoglu is currently resident in cardiology at Mehmet Akif Ersoy Hospital in Turkey. He completed his medical school training at the Trakya University in 2022. His field of interest is invasive cardiology.

## Data Availability

Data used in this paper are available to readers upon request. The authors confirm that the data supporting the findings of this study are available within the article.

## Notes

**Consent:** Informed consent was obtained from the patient in accordance with COPE guidelines.

**Funding:** None declared.
